# Behaviors of consumers, physicians and pharmacists in response to adverse events associated with dietary supplement use

**DOI:** 10.1186/s12937-017-0239-4

**Published:** 2017-03-18

**Authors:** Tsuyoshi Chiba, Yoko Sato, Etsuko Kobayashi, Kazuki Ide, Hiroshi Yamada, Keizo Umegaki

**Affiliations:** 1 Information Center, National Institute of Health and Nutrition, National Institutes of Biomedical Innovation, Health and Nutrition, 1-23-1 Toyama, Shinjuku-ku, Tokyo 162-8636 Japan; 2 Department of Drug Evaluation & Informatics, Graduate School of Pharmaceutical Sciences, University of Shizuoka, 52-1 Yada, Suruga-ku, Shizuoka 422-8526 Japan

**Keywords:** Dietary supplements, Adverse events, Consumers, Physicians, Pharmacists

## Abstract

**Background:**

The prevalence of dietary supplements has increased in Japan, and, as a consequence, the adverse events associated with dietary supplement use have become more prominent. Severe adverse events must be reported to the Japanese government via public health centers. However, the number of cases reported to the Japanese government is limited. To clarify this discrepancy, we conducted an internet questionnaire, and surveyed how consumers, physicians and pharmacists acted when they or their patients developed adverse events due to dietary supplement use.

**Methods:**

This study was completed by 2732 consumers, 515 physicians, and 515 pharmacist via internet surveillance on November 2015.

**Results:**

Although 8.8% of consumers developed adverse events including diarrhea, constipation, stomachache, headache, and nausea and vomiting, most of them did not report their adverse events to public health centers. However, some consumers went to hospitals because of adverse events. We also surveyed how physicians and pharmacists acted when their patients developed adverse events due to dietary supplement use. Most physicians and pharmacists did not report these cases to public health centers because they were unable to definitively prove the cause-and-effect relationship of these adverse events. Furthermore, some physicians and pharmacists did not know how or where to report these adverse events.

**Conclusions:**

We clarified the reasons for the limited number of reports of adverse events to the Japanese government in this survey. It is important to encourage not only consumers, but also physicians and pharmacists to report adverse events to public health centers. In addition, an analyzing tool of cause-and-effect relationships might be helpful for physicians and pharmacists.

**Electronic supplementary material:**

The online version of this article (doi:10.1186/s12937-017-0239-4) contains supplementary material, which is available to authorized users.

## Background

The prevalence of dietary supplements, which are helpful to supply nutrients for health, has increased in Japan [1–3]. Consumers use not only vitamins and minerals, but also various other ingredients as dietary supplements. However, most consumers do not understand the properties of dietary supplements and their ingredients, which may be beneficial, but are also associated with a risk of adverse reactions, such as gastrointestinal and neurological complications, hepatotoxicity, and interactions with drugs [4–8]. The reasons for misunderstanding of consumers are that manufacturers tend to emphasize the key characteristics of their products and promote sales using attractive claims [9]. Furthermore, some dietary supplements have claimed beneficial effects for the treatment of several diseases, even though these claims are illegal in Japan. Under these conditions, consumers use dietary supplements inappropriately, thereby increasing the risk of adverse events.

There are several causes of adverse events due to dietary supplement use, the first of which is the use of low quality dietary supplements. For example, an excess amount of heavy metals or unlabeled ingredients associated with allergies may contaminate products. Drug ingredients, such as sibutramine, sildenafil, and dexamethasone, are added to dietary supplements [10, 11]. A large number of studies have reported a relationship between adverse events and the use of these products. Another cause of adverse events is consumer characteristics. Some individuals develop allergic reactions to the ingredients of dietary supplements. Infants/children, pregnant and lactating women, and the elderly are influenced more by dietary supplements [12, 13]. In addition, patients, particularly those receiving medication, need to be more cautious of dietary supplement use [14]. A third cause of adverse events is the inappropriate use of dietary supplements including an excessive intake or the concomitant use of various dietary supplements and/or medicines. In the past few decades, a large number of ingredients have been shown to interact with drugs [15–21]; however, it currently remains unclear whether most ingredients in dietary supplements interact with drugs.

We previously reported that some dietary supplement users in Japan experienced adverse events [22, 23]. There are several ways by which to report the adverse events associated with dietary supplement use in Japan. Consumers report their adverse events to manufacturers or retail stores. In the US, dietary supplements are regulated by the Dietary Supplement Health and Education Act (DSHEA) [24, 25]. Under the DSHEA, the manufacturers, packers, and distributors of dietary supplements are required to submit the main associated adverse events to the US Food and Drug Administration (FDA)’s Center for Food Safety and Applied Nutrition Adverse Event Reporting System (CAERS) within 15 business days of receiving information on adverse events. On the other hand, manufacturers do not have a duty to report adverse events to the Ministry of Health, Labour and Welfare (MHLW) in Japan, which regulates adverse events associated with dietary supplement use. Therefore, the majority of complaints are handled by each manufacturer. Consumers also have the option of reporting adverse events to the Practical Living Information Online Network System (PIO-NET), which is the operating system of the National Consumer Affairs Center of Japan. Several hundred cases per year are reported to the PIO-NET [26]. However, most reports cannot define cause-and-effect relationships because the information obtained from consumers is not considered to be sufficient to define these relationships. Furthermore, consumers themselves or their physicians or pharmacists report severe adverse events associated with dietary supplement use to public health centers, which then report adverse events to the MHLW. However, only 20 adverse events per year are reported to the MHLW. Therefore, the Japanese government cannot take action against adverse events associated with dietary supplement use. The cause of the limited number of adverse events reported to the MHLW currently remains unclear.

In the present study, we surveyed how consumers behaved when they experienced adverse events associated with dietary supplement use. We also surveyed how physicians and pharmacists advised their patients and whether they reported adverse events to public health centers.

## Methods

### Definition of “dietary supplement”

In Japan, “dietary supplement” has no definition in the law, and some dairy or soybean products are recognized as dietary supplements even if they are in the form of common foods. Therefore, the meaning of the term “dietary supplement” differs for each individual. In this survey, “dietary supplement” was defined as those that consumers considered to have beneficial effects on their health (e.g., vitamins, minerals, fish oil, glucosamine, cereal) except for daily foods (e.g., vegetables, fruits, milk, tofu).

### Internet survey

An internet-based questionnaire was conducted by Macromill, Inc. (Tokyo, Japan) with their research registrants (2 million registrants in 2015) between 13 November and 19 November 2015 on consumers, and between 13 November and 18 November 2015 on physicians and pharmacists. This study was conducted with the approval of the Research Ethics Committee of the National Institutes of Biomedical Innovation, Health and Nutrition (No.198-1, approved on Aug. 28, 2015) and in accordance with Declaration of Helsinki.

### Preliminary survey for subjects who experienced adverse effects due to dietary supplement use

To extract consumers who experienced adverse events due to dietary supplement use, a preliminary survey was conducted with randomly selected 70,000 consumers from research registrants of Macromill, Inc. (Additional file 1). Of these, 24,295 subjects (34.7%) were currently using dietary supplements, 18,194 (26.0%) had used dietary supplements in the past, and 27,511 (39.3%) have never used dietary supplements. We asked current- and ex-users of dietary supplements whether they had experienced adverse events (diarrhea, constipation, stomachache, headache, nausea and vomiting, fatigue, anthema and itching, palpitations, worsening health examination data, or other symptoms) due to dietary supplement use. A total of 6129 subjects (8.8%) experienced adverse events, and a part of these consumers answered the full survey. Veterinary surgeons and dentists were excluded from the survey because they had few opportunities to be asked by patients about the adverse events associated with dietary supplement use.

### Full survey

The full survey was conducted on 3095 consumers who experienced adverse events (Additional file 2). The questionnaire included the purpose of supplementation (maintenance of health, improvements to health, for beauty or weight loss, prevention of diseases, and treatment of diseases), types of dietary supplements, their responses to adverse events which associated by dietary supplement use, and their reasons if they did not report it to public institutes. Among physicians and pharmacists, the survey was conducted on 1030 subjects (515 physicians and 515 pharmacists) (Additional file 3). The questionnaire included their experiences of being asked by patients about adverse events associated with dietary supplement use, what advice they gave their patients, whether they reported it to public institutes, and their reasons if they did not report it to public institutes. The demographic characteristics (sex and age) were obtained from the research registrants’ data of Macromill, Inc.

### Statistical analysis

Differences among groups were tested using the *χ* ^2^ test. Statistical analyses were performed using HALBAU 7.5.1 (HALBAU R&D Co., Ltd., Tokyo). *P*-values less than 0.05 were considered significant.

## Results

### Characteristics

Characteristics of consumers, physicians and pharmacists are shown in Table 1. Consumers (*n* = 3095) were as follows: sex (male; 1340, female; 1755), age (20s; 468, 30s; 736, 40s; 634, 50s; 611, older than 60; 646). The characteristics of physicians and pharmacists (*n* = 1030) were as follows: sex (female; 379, male; 651), age (20s; 64, 30s; 248, 40s; 317, 50s; 289, older than 60; 112). Physicians (515) specialized in internal medicine; 214, pediatrics; 34, obstetrics and gynecology; 18, and others; 249. Pharmacists (515) worked in dispensing pharmacies; 416, drugstores; 24, and others; 75.

**Table 1:** Characteristics of consumers, physicians and pharmacists

	Consumers		Physicians		Pharmacists	
	n	%	n	%	n	%
All	3095		515		515	
Sex						
Male	1340	43.3	440	85.4	211	41.0
Female	1755	56.7	75	14.6	304	59.0
Age						
20–29	468	15.1	10	1.9	54	10.5
30–39	736	23.8	71	13.8	177	34.4
40–49	634	20.5	171	33.2	146	28.3
50–59	611	19.7	192	37.3	97	18.8
≥ 60	646	20.9	71	13.8	41	8.0

### Purpose of dietary supplement use

The purposes indicated by consumers for the use of dietary supplements are shown in Table 2. The main reason that consumers used dietary supplement was for the maintenance of health. Other reasons were for improvements to health, for beauty, the prevention of diseases, weight loss, and the treatment of disease. The prevalence of the prevention of disease, weight loss, and treatment of disease was significantly higher in subjects who experienced adverse events in the full survey than in all subjects in the preliminary survey. This result suggests that dietary supplements for these purposes cause more adverse events.

**Table 2:** Purpose of dietary supplement use

	AE^a^		All^b^	
	n	%	n	%
Maintenance of health	2336	75.5	33,347	78.5
Improvements to health	936	30.2	9500	22.4
For beauty	841	27.2	9977	23.5
Prevention of diseases	742	24.0	7906	18.6
Weight loss	738	23.8	7014	16.5
Treatment of diseases	222	7.2	1714	4.0
Others	63	2.0	821	1.9

^a^subjects who experienced adverse events (*n* = 3095)

^b^all subjects who were using or used to use dietary supplement in preliminary survey (*n* = 42,489)

In addition, ratio of who used dietary supplement to treat their diseases was greater in subject who experienced adverse events (7.2%) compared to all subject (4.0%). This data suggests that adverse events were more frequently occurred, when they used dietary supplements to treat their diseases.

### Symptoms of adverse events

The symptoms of adverse events due to dietary supplement use are shown in Table 3. Diarrhea was the most frequent adverse event, followed by nausea and vomiting, fatigue, and constipation. Diarrhea, nausea and vomiting, headache, and stomachache were significantly more prevalent in younger consumers than in the elderly. On the other hand, anthema and itching and worsening health examination data were significantly more prominent in the elderly than in younger consumers.

**Table 3:** Adverse events due to dietary supplement use (*n* = 3095)

	All (%)	20s (%)	30s (%)	40s (%)	50s (%)	Older than 60 (%)	*P*-value
Diarrhea	27.9	35.0	32.7	29.3	27.3	16.4	<0.001
Nausea & vomiting	19.1	23.7	22.8	21.3	17.3	11.0	<0.001
Fatigue	17.4	19.7	17.4	18.6	15.4	16.3	0.327
Constipation	17.0	17.7	16.4	15.6	15.5	20.0	0.189
Headache	15.6	22.4	19.0	15.9	13.7	8.4	<0.001
Stomachache	15.4	23.1	17.7	15.1	13.3	9.4	<0.001
Anthema & itching	15.0	11.8	13.0	14.8	14.1	20.6	<0.001
Worsening data of health examination	10.2	4.3	4.1	8.2	11.9	22.0	<0.001
Palpitations	5.5	4.5	5.7	5.7	5.6	5.7	0.895
Others	11.6	9.6	9.4	11.0	13.1	14.9	0.009

Statistical analysis were conducted among ages in each event. *P*-values were calculated using the *χ* ^2^ test

### Details of dietary supplements potentially associated with adverse events

Details of dietary supplements potentially associated with adverse events are shown in Table 4. Among vitamins/minerals, multi-vitamins were more frequently associated with adverse events than multi-minerals or multi-vitamins and minerals. Individual mineral, such as Fe, Zn, and Ca, and individual vitamin, including vitamin B, vitamin C, and vitamin E also caused adverse events. Furthermore, various non-vitamin, non-mineral supplements were associated with the development of adverse events. *Coleus forskohlii*, which is one of the popular ingredients used to promote weight loss in Japan, was the top ingredient that caused adverse events at a rate that was more than two-fold that of glucosamine/chondroitin, garlic, sesamin, collagen, and other ingredients. In addition, weight loss supplements (except for *Coleus forskohlii*) were strongly associated with the development of adverse events.

**Table 4:** Details of dietary supplements potentially causing adverse events

	n	Percent
Vitamin/Mineral		
Multi-vitamins	117	3.8
Multi-minerals	20	0.6
Multi-vitamins and minerals	15	0.5
Individual vitamin (vitamin B, vitamin C, and vitamin E)	162	5.2
Individual mineral (Fe, Zn, and Ca)	214	6.9
Non-Vitamin, Non-Mineral (Top 10)		
*Coleus forskohlii*	183	5.9
Glucosamine/Chondroitin	87	2.8
Garlic	70	2.3
Sesamin	69	2.2
Collagen	64	2.1
Blueberry/Lutein	53	1.7
EPA/DHA/Fish oil/n-3 PUFA	50	1.6
Placenta	44	1.4
Enzymes	42	1.4
Royal jelly/Propolis	41	1.3
Weight loss supplements (except for *Coleus forskohlii*)	155	5.0

### Responses of consumers to adverse events

Responses of consumers to adverse events are shown in Table 5. Almost half of consumers stopped using dietary supplements immediately. Adverse events are typically ameliorated by the discontinuation of dietary supplements. On the other hand, one-third of consumers did nothing. Only 5.8% of consumers reported adverse events to public institutes (5.1% to public health centers, 0.5% to the National Consumer Affairs Center of Japan or other consumer affairs centers, and 0.2% to the MHLW or Consumer Affairs Agency, Government of Japan). The reason why most consumers did not report to public institutes was “adverse events were not sufficiently severe to make a complaint (70.8%)”. Other reasons were “there were other possibilities for adverse events (28.1%)” or “making a complaint to public institutes was considered to be too troublesome (13.5%)”.

**Table 5:** Responses of consumers to adverse events

	n	Percent
Stopped using dietary supplements immediately	1669	53.9
Did nothing	1164	37.6
Went to a hospital	184	5.9
Complained to manufacturers	167	5.4
Reported to public health centers	159	5.1
Complained to the retail store	42	1.4
Reported to the National Consumer Affairs Center of Japan or other consumer affairs centers	16	0.5
Reported to the MHLW or Consumer Affairs Agency, Government of Japan	6	0.2

*MHLW* Ministry of Health, Labour and Welfare

A total of 5.9% of consumers who experienced adverse effects went to a hospital. Physicians and pharmacists have a responsibility to report adverse events associated with dietary supplement use to public health centers.

### Consultations regarding adverse events

We conducted a survey on physicians and pharmacists regarding consultations related to dietary supplements. The 57.3% (295/515) of physicians and 59.0% (304/515) of pharmacists were asked about dietary supplement use by their patients. Of these, 25.4% (131/515) of physicians and 19.6% (101/515) of pharmacists had consultations on adverse events with their patients (Table 6). Physicians had significantly more opportunities for consultations on adverse events as compared to pharmacists. Most consultations only occurred 1–2 times, whereas some were more than 3 times. A total of 11.5% (15/131) of physicians and 6.9% (7/101) of pharmacists had more than 10 consultations.

**Table 6:** Opportunity of consultations related to adverse reactions with their patients

	Physicians		Pharmacists		
	n	%	n	%	*P*-value
Yes, I have.	131	25.4	101	19.6	0.030
No, I have never.	384	74.6	414	80.4	
Number of times					
1–2	75	14.6	69	13.4	0.081
3–5	38	7.4	19	3.7	
6–9	3	0.6	6	1.2	
More than 10	15	2.9	7	1.4	

Physicians (*n* = 515), Pharmacists (*n* = 515), *P*-values were calculated using the *χ* ^2^ test

### Responses of physicians and pharmacists to adverse events among their patients

Among physicians and pharmacists who had consultations on adverse events, most physicians (84.0%) and pharmacists (75.2%) advised their patients to stop using dietary supplements immediately (Table 7). On the other hand, only 12.3% of physicians and 17.5% pharmacists reported these events to public institutes (8.4 and 11.9% to public health centers, 3.1 and 5.9% to the National Consumer Affairs Center of Japan or other consumer affairs centers, and 0.8 and 1.0% to the Consumer Affairs Agency, Government of Japan). There were no significant differences in behaviors between physicians and pharmacists, except for ask manufactures. Pharmacists (18.8%) significantly more frequently asked manufactures about their products compared to Physicians (3.8%).

**Table 7:** Responses of physicians and pharmacists to adverse events among their patients

	Physicians		Pharmacists		
	n	%	n	%	*P*-value
Advice to stop using dietary supplements immediately	110	84.0	76	75.2	0.137
Nothing apart from a follow-up	32	24.4	24	23.8	1.000
Ask patients to report to other institutes by themselves	14	10.7	19	18.8	0.117
Ask manufacturers about their products	5	3.8	19	18.8	<0.001
Report to public health centers	11	8.4	12	11.9	0.510
Report to the National Consumer Affairs Center of Japan or other consumer affairs centers	4	3.1	6	5.9	0.455
Report to the Consumer Affairs Agency, Government of Japan	1	0.8	1	1.0	-
Others	4	3.1	3	2.3	-

Physicians (*n* = 131), Pharmacists (*n* = 101), *P*-values were calculated using the *χ* ^2^ test

### Reasons why physicians and pharmacists did not report adverse events to public health centers

The reasons why physicians and pharmacists did not report adverse events to public health centers were similar (Table 8). They were unable to define case-and-effect relationships for adverse events. Furthermore, they did not know what level of severity needed to be reported. These two reasons were significantly higher in pharmacists than physicians. On the other hand, one third of physicians and pharmacists did not know to where to report adverse events. This reason was significantly higher in physicians than pharmacists.

**Table 8:** Reasons why physicians and pharmacists did not report adverse events to public health centers

	Physicians		Pharmacists		
	n	%	n	%	*P*-value
It is difficult to define a cause-and-effect relationship	284	55.1	343	66.6	<0.001
It is not clear which case (severity) needs to be reported	214	41.6	249	48.3	0.033
It is not clear where to report	224	43.5	172	33.4	0.001
Cumbersome procedure to report	61	11.8	51	9.9	0.368
Others	18	3.5	11	2.1	0.258

Physicians (*n* = 515), Pharmacists (*n* = 515), *P*-values were calculated using the *χ* ^2^ test

## Discussion

We previously reported that some patients used dietary supplements to treat their diseases [22], and concomitantly used dietary supplements and medicines [23]. The inappropriate usage of dietary supplements causes adverse events. The percentages of consumers who experienced adverse events were 3.3 and 8.4% in each report. Several hundred complaints about adverse events due to dietary supplement use are reported to the National Consumer Affairs Center of Japan or other consumer affairs centers every year [26]. However, our results showed that most consumers, physicians, and pharmacists did not report adverse events to public health centers. This is the main reason why the number of adverse events reported to the MHLW is limited.

In the US, dietary supplements are regulated by the DSHEA; however, the DSHEA does not work well. The FDA almost daily warns or recalls tainted dietary supplements, such as the sexual enhancement products containing sildenafil, tadalafil, or their analogues, the weight-loss products containing sibutramine and the laxative phenolphthalein, and the sports supplements containing 1,3-dimethylamylamine or anabolic steroids. On the other hand, manufacturers have to report adverse events to the FDA’s CAERS [24, 25]. However, inadequate and poor-quality reports on adverse events associated with dietary supplements are the main limitations of safety monitoring [4, 27]. This condition is similar to Japan. Although several hundred cases per year are reported to the PIO-NET from consumers [26], most reports are inadequate and of insufficient quality to define cause-and-effect relationships. On the other hand, the number of adverse event reports from physicians and pharmacists is limited, but are based on clinical examinations. Therefore, it is important to increase the number of adverse event reports from physicians and pharmacists. However, some physicians and pharmacists have inadequate knowledge about dietary supplements and do not know how or where to report adverse events [28, 29]. Furthermore, 28.0% of physicians and 35.1% of pharmacists were using dietary supplements themselves, while 20.6% of physicians and 35.1% of pharmacists had previously used dietary supplements in this present survey. This result indicates that half of physicians and pharmacists might have a positive attitude towards dietary supplements and may pay less attention to the adverse events associated with their use.

There are some reports that most of patients did not inform their physicians/pharmacists about dietary supplement use, and communication between patients and physicians/pharmacists was important to avoid adverse events [22, 30]. On the other hand, there is no report whether there are differences in attitude towards dietary supplement use in their patients between physicians and pharmacists, and whether physicians and pharmacists discuss dietary supplement use in their patients each other. In our survey, there were some differences between physicians and pharmacists in opportunities for consultation, management of adverse events, and their own personal supplement use. In this regard, communication between physicians and pharmacists might be an important strategy to prevent adverse events and to increase the number of adverse event reports to public health centers.

Our survey also clarified the features of adverse events among generations. This difference might be attributed to the different types of dietary supplements used by each generation. The prevalence of anthema and itching was significantly higher among the elderly, who prefer to use glucosamine/chondroitin for arthralgia [31]. Glucosamine/chondroitin have been shown to induce an allergic reaction [32]. On the other hand, the prevalence of diarrhea was significantly higher in the younger generation, which prefers to use dietary supplements for weight loss. In Japan, most young women want to lose weight in order to maintain their appearance, even though being excessively thin is an important issue among this demographic [33]. A large number of dietary supplements for weight loss contain *Coleus forskohlii*, *Red pepper*, *Senna*, or other ingredients that may cause diarrhea [34–36]. However, it is difficult to define cause-and-effect relationships among them. A generation difference has also been reported in adverse events in the US [13]. Adverse events were associated with dietary supplements for weight loss or energy products in younger generations, and with micronutrients in older generations. Therefore, appropriative information to avoid adverse events are needed for each generation.

There are several limitations to the passive collection of adverse reactions associated with dietary supplements. Consumers, physicians, and pharmacists do not report to public health centers if adverse events are not severe. Furthermore, the numbers of reported adverse reactions that define cause-and-effect relationships are very low. Therefore, the MHLW cannot establish whether these cases are common or singular. However, a one-year prospective surveillance study on dietary supplement-related poison control center calls enabled the rapid detection of potentially harmful products [37]. In this regard, the active collection of adverse events associated with dietary supplements, such as an internet questionnaire, may be a useful method for the rapid detection of harmful products because a large number of consumers obtain information on dietary supplements via the internet [38]. In addition, 23.5% of consumers concomitantly used dietary supplements and medicines in this survey. There is a possibility that interaction of dietary supplements and medicines might cause adverse events. However, we could not estimate how it contributed to our results, because we did not ask in detail about their medications.

In this survey, we clarified that most of consumers did not report adverse events by their self, and physicians and pharmacists also did not report, because they could not define cause-and-effect relationships. So, the number of reports on adverse events to the MHLW are limited. There are two possible ways by which to improve this limitation. A large number of physicians and pharmacists are unable to define case-and-effect relationships for adverse events. In order to resolve this issue, easy-to-use assessment methods (a modified Naranjo scale and modified FDA algorithm) may be useful [39]. These methods enable cause-and-effect relationships for adverse events to be determined. Furthermore, although some physicians and pharmacists reported adverse events to public health centers, public health centers may not report adverse events to the MHLW. Public health centers may receive a large number of reports that include several levels of adverse events. Public health centers review these reports and only report severe adverse events to the MHLW. Therefore, a survey on public health centers is needed in order to clarify the extent to which public health centers receive reports on adverse events and what they report to the MHLW.

## Conclusions

Our investigation revealed that a large number of consumers experienced adverse events due to dietary supplement use; however, most of these events were not severe. Although some consumers went to a hospital because of adverse events, physicians and pharmacists were unable to define cause-and-effect relationships for adverse events. Therefore, the reconsideration of a reporting system is needed in order for the MHLW to collect reports on adverse events. It is important that not only patients, but also physicians and pharmacists actively report adverse events to public health centers to decrease and prevent adverse events due to dietary supplement use.

## Additional files

Additional file 1: Preliminary Survey for Consumers. (DOCX 15 kb)
Additional file 2: Full Survey for Consumers. (DOCX 15 kb)
Additional file 3: Survey for Physicians and Pharmacists. (DOCX 15 kb)

## Abbreviations

DHA: Docosahexaenoic acid
DSHEA: Dietary Supplement Health and Education Act
EPA: Eicosapentaenoic acid
FDA: Food and Drug Administration
MHLW: Ministry of Health, Labour and Welfare
PUFA: Polyunsaturated fatty acid
