# Supplementary material for: Behaviors of consumers, physicians and pharmacists in response to adverse events associated with dietary supplement use
Source: Nutr J. 2017 Mar 18;16:18. doi: 10.1186/s12937-017-0239-4 (PMC5357328; doi:10.1186/s12937-017-0239-4)
Supplement: Additional file 1: — Preliminary Survey for Consumers. (DOCX 15 kb) [file 12937_2017_239_MOESM1_ESM.docx]

**Additional file 1**

**Preliminary Survey for Consumers**

Q1: At present, are you using any dietary supplements?

A1: Yes, I am using dietary supplements.

A2: No. I used to use dietary supplements, but I quitted.

A3: No, I have never used.

People who answered A1 or A2 moved to Q2.

Q2: Which purpose are/were you using dietary supplements for? (Multiple choice)

A1: maintenance of health

A2: improvements to health

A3: for beauty

A4: weight loss

A5: prevention of diseases

A6: treatment of diseases

A7: others

Q3: Have you ever experienced any adverse effects by using dietary supplements? (Multiple choice)

A1: No, I have not.

A2: nausea and vomiting

A3: headache

A4: stomachache

A5: diarrhea

A6: constipation

A7: anthema &, itching

A8: fatigue

A9: palpitations

A10: worsening data of health examination (ex. fasting blood glucose, lipids level, or blood pressure)

A11: others

A part of people who answered from A2 to A11 on Q3 moved to “Full Survey”.
