# Supplementary material for: Behaviors of consumers, physicians and pharmacists in response to adverse events associated with dietary supplement use
Source: Nutr J. 2017 Mar 18;16:18. doi: 10.1186/s12937-017-0239-4 (PMC5357328; doi:10.1186/s12937-017-0239-4)
Supplement: Additional file 2: — Full Survey for Consumers. (DOCX 15 kb) [file 12937_2017_239_MOESM2_ESM.docx]

Additional file 2

**Full Survey for Consumers**

Q1: Do you remember which dietary supplements were you using when you experienced adverse events?

And if yes, answer the name of products that you used.

A1: Yes.

A2: No

Q2. How did you deal with the adverse event? (Multiple choice)

A1: Did nothing

A2: Stopped using dietary supplement immediately

A3: Complained to manufacturers

A4: Complained to the retail store

A5: Reported to the National Consumer Affairs Center of Japan or other consumer affairs centers

A6: Reported to the MHLW or Consumer Affairs Agency, Government of Japan

A7: Reported to public health centers

A8: Went to a hospital

A9: Others

Q3: Do you know that you should report to public health centers when you experienced adverse events by dietary supplement use?

A1: No.

A2: Yes, I do. I reported to public health centers.

A3: Yes, I do. But I did not report to public health centers.

A part of people who answered A3 moved to Q4.

Q4: Why didn’t you report to public health centers?

A1: I thought that adverse event was not severe.

A2: There were some possibilities other than dietary supplement use.

A3: It was cumbersome to report.

A4: I did not know how to report.

A5: I did report to other agencies.

A6: Others
