# Supplementary material for: Behaviors of consumers, physicians and pharmacists in response to adverse events associated with dietary supplement use
Source: Nutr J. 2017 Mar 18;16:18. doi: 10.1186/s12937-017-0239-4 (PMC5357328; doi:10.1186/s12937-017-0239-4)
Supplement: Additional file 3: — Survey for Physicians and Pharmacists. (DOCX 15 kb) [file 12937_2017_239_MOESM3_ESM.docx]

**Additional file 3**

**Survey for Physicians and Pharmacists**

Q1: Have you ever have opportunities to consult with your patients about dietary supplement use?

A1: Yes.

A2: No

Q2: Have you ever have opportunities to consult with your patients about adverse events associated to dietary supplement use?

And if yes, how many times have you ever have opportunities?

A1: 1~2

A2: 3~5

A3: 6 - 9

A4: More than 10

Q3. How did you deal with your patients who developed adverse event? (Multiple choice)

A1: Nothing apart from a follow-up

A2: Advice to stop using immediately

A3: Ask patients to report to other institutes by themselves

A4: Ask manufacturers

A5: Reported to the National Consumer Affairs Center of Japan or other consumer affairs centers

A6: Report to the Consumer Affairs Agency, Government of Japan

A7: Reported to public health centers

A8: Others

Q4: Why didn’t you report to public health centers? (Multiple choice)

A1: I thought that adverse event was not severe.

A2: There were some possibilities other than dietary supplement use.

A3: It was cumbersome to report.

A4: I did not know how to report.

A5: I did report to other agencies.

A6: Others
